# Implications of LDH in patients with coronavirus disease 2019 pneumonia

**DOI:** 10.3389/fcimb.2023.1180187

**Published:** 2023-10-30

**Authors:** Tong Mu, Xingguang Wang, Zhiming Lu, Jia Tong

**Affiliations:** ^1^ Department of Gastroenterology, Shandong Provincial Hospital Affiliated to Shandong First Medical University, Shandong First Medical University, Jinan, Shandong, China; ^2^ Department of Respiratory, Shandong Provincial Hospital Affiliated to Shandong First Medical University, Shandong First Medical University, Jinan, Shandong, China; ^3^ Department of Clinical Laboratory Medicine, Shandong Provincial Hospital Affiliated to Shandong First Medical University, Shandong First Medical University, Jinan, Shandong, China; ^4^ Department of Geriatric Medicine, Shandong Provincial Hospital Affiliated to Shandong First Medical University, Shandong First Medical University, Jinan, Shandong, China

**Keywords:** COVID-19, LDH, pneumonia, progression, neutrophil

## Abstract

**Objective:**

The objective of this study was to explore the value of serum lactic dehydrogenase (LDH) in the early diagnosis and prognostic evaluation of pneumonia associated with the novel coronavirus infection.

**Methods:**

A total of 101 patients with coronavirus disease 2019 (COVID-19) pneumonia were included in the study. According to the severity of the initial chest computed tomography (CT), the patients were divided into the ordinary pneumonia group and the severe pneumonia group and then divided into the remission group and the nonremission group according to the changes of the chest CT after medication treatment. The differences in general characteristics, underlying diseases, clinical symptoms, laboratory findings, and imaging examination outcomes between groups were observed retrospectively. To analyze the diagnostic performance of LDH, receiver operating characteristic (ROC) curves were constructed and the area under the curve (AUC) was calculated.

**Results:**

Compared with ordinary pneumonia patients, patients in the severe group presented with significantly higher LDH, neutrophil count, high-sensitivity troponin T (HS-TnT), C-reactive protein (CRP), human serum amyloid A (SAA), N-terminal pro-brain natriuretic peptide (NTproBNP), and D-dimer. Compared with remission patients, non-remission patients presented with significantly higher LDH, neutrophil count, HS-TnT, CRP, SAA, procalcitonin (PCT), creatine kinase–MB mass (CKMB_M), NTproBNP, and D-dimer. In multivariate logistic regression analysis, we found that LDH [odds ratio (OR), 1.015; 95% confidence interval (CI), 1.006–1024; *p* = 0.001] and neutrophil count (OR, 1.352; 95% CI, 1.008–1.811; *p* = 0.044) were independently associated with exacerbation in COVID-19 patients. For ROC analysis, the AUC was 0.833 (95% CI, 0.729–0.936; *p* < 0.001) when we use the LDH value of 256.69 U/L to discriminate the ordinary pneumonia and severe pneumonia patients. The AUC was 0.759 (95% CI, 0.603–0.914; *p* = 0.008) and the sensitivity is 92.3% when we combined the LDH (cutoff value 258.46 U/L) and the neutrophil count (cutoff value 6.76 × 10^9^/L) to discriminate remission and non-remission patients.

**Conclusion:**

The level of LDH is associated with the severity of COVID-19 pneumonia and can be used as important indicators to evaluate the prognosis of patients.

## Introduction

Coronavirus disease 2019 (COVID-19) is still spreading globally since December 2019. In mid-December 2022, an epidemic of COVID-19 infections spread rapidly through China. The clinical manifestations of patients with COVID-19 infection are diverse, including fever, cough, sore throat, chest tightness, diarrhea, fatigue, depression, loss of appetite, shortness of breath, and decreased oxygen saturation (Kevadiya et al., 2021). Patients in the progressive phase of COVID-19 may need invasive auxiliary ventilation, even death because of severe acute respiratory syndrome (ARDS) or multiple organ failure (Wu et al., 2017; Huang et al., 2020). It is particularly important to accurately judge the trend of the change of the condition in COVID-19 patients and carry out relevant treatment programs as soon as possible. By retrospectively analyzing the differences between clinical symptoms, laboratory test results, and chest computed tomography (CT) outcomes of COVID-19 patients with ordinary pneumonia or severe pneumonia, we wanted to explore the relevant indicators that can evaluate the severity of pneumonia and predict the progress of COVID-19 pneumonia and to take intervention measures timely that can significantly improve the prognosis of patients and reduce the occurrence of case fatality rate. Abnormal coagulation function and increase of D-dimer level (Libby, 2020) may be related to the severe poor prognosis and death of COVID-19 patients. A previous study has reported that a high level of lactic dehydrogenase (LDH) is related to respiratory function and a predictor of respiratory failure in community-acquired pneumonia (CAP) patients (Zhong-shu Kuang and Sun, 2020). Whether LDH is a risk factor for determining the severity and prognosis of COVID-19 has not been reported.

We conducted this retrospective study to compare levels of biomarkers among COVID-19 patients and found that the level of LDH was associated with the severity of COVID-19 pneumonia and can be used as important indicators to evaluate the prognosis of patients. We assist clinicians in monitoring and evaluating the severity and prognosis of COVID-19 and provide guidance for the treatment of COVID-19 patients.

## Methods

### Study design and participants

In this single-center retrospective observational study, we retrospectively included adult patients with COVID-19 who were admitted to the department specializing in COVID-19 treatment from 12 December 2022 to 20 January 2023. The inclusion criteria were as follows: 1) age ≥18 years; 2) patients with positive COVID-19 nucleic acid testing by real-time quantitative reverse transcription polymerase chain reaction (RT-qPCR); 3) patients undergoing the first chest CT scans in our hospital, which showed viral pneumonia. The exclusion criteria were as follows: 1) patients of SARS-CoV-2 infection complicated with other common respiratory pathogenic microorganisms; 2) patients suffering from acute myocardial infarction or end-stage renal disease on admission. The ethics committee of our hospital approved this study and granted a waiver of informed consent from participants.

### Detection of SARS-CoV-2 and other pathogens

Nasopharyngeal swabs were collected from the patients within 48 h before hospitalization or at admission. An RT-qPCR assay was performed to detect viral RNA using the commercialized nucleic acid detection kits (Sansure Biotech Inc.). A cycle threshold (Ct) value less than 40 was considered positive for SARS-CoV-2. A confirmed case of COVID-19 was defined if the patient had a positive RT-qPCR test result. Peripheral blood samples were collected at admission, and the serum IgM antibody of human respiratory syncytial virus, adenovirus, influenza A virus (IAV), influenza B virus (IBV), human parainfluenza virus, *Chlamydia* pneumoniae, Coxsackie B virus, enteric cytopathic human orphan (ECHO) virus, and *Mycoplasma* pneumoniae was tested using the corresponding IgM antibody detection kit (Beier Bioengineering Co., Ltd.) by magnetic particle chemiluminescence method. Respiratory bacteria and fungi were detected by sputum smear and culture.

### Outcomes

Two outcomes were evaluated: severity of pneumonia shown on the first chest CT and the change of pneumonia shown on the second chest CT. Patients were divided into the ordinary pneumonia group and the severe pneumonia group according to the first chest CT and the pneumonia remission group and non-remission group according to the latter outcome. All chest CT findings were evaluated by physicians and radiologists who were blinded to the clinical data.

**Figure 1:**
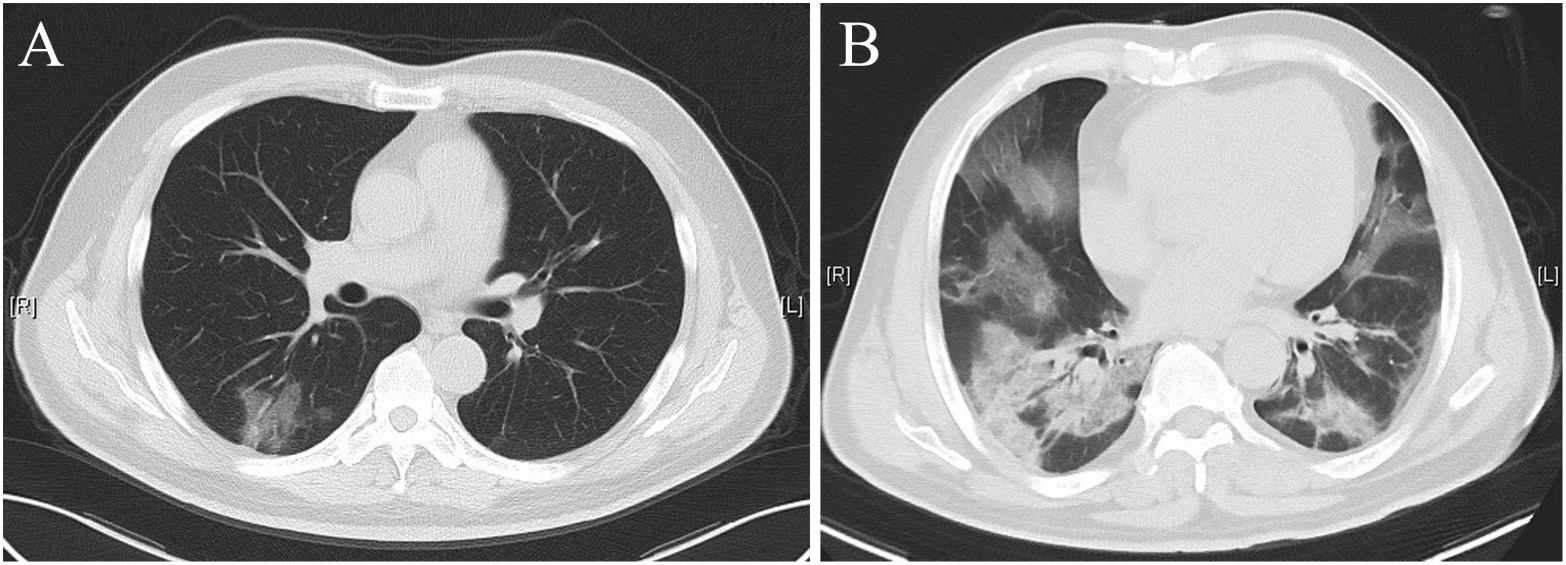
Chest computed tomography (CT) scan of the ordinary group **(A)** and the severe group **(B)**.

Chest CT features of the ordinary pneumonia group contain the following: limited lesions with patchy subsegmental or segmental distribution; lesions are predominantly distributed in the lateral segment of the lung or subpleural region; single or multiple ground‐glass opacities; air bronchogram; vascular enlargement (Li K. et al., 2020) (**Figure 1A**).

Chest CT features of the severe pneumonia group contain the following: involvement of multiple lobes; diffuse lesions in bilateral lung; extensive exudation and consolidation, mainly consolidation; traction bronchiectasis; crazy-paving pattern; interlobular septal thickening; lymph node enlargement; pleural effusion (Li K. et al., 2020; Zheng et al., 2021) (**Figure 1B**).

Chest CT features indicating remission of pneumonia contain the following: pneumonia resolution completely or partially without new lesion; pneumonia resolution partially with new focal lesion; increased density of the original lesion and reduced inflammatory exudation around the original lesion. Patients with opposite imaging findings are included in the non-remission group.

### Statistical analysis

Continuous variables are expressed as mean [standard deviation (SD)] and median [interquartile range (IQR)]. The means for continuous variables were compared using independent group t-tests when the data accorded with normal distribution; otherwise, the Mann–Whitney–Wilcoxon test was used. Categorical variables are shown as number (%) and were compared using the χ^2^ test or the Fisher exact test. The changes in laboratory parameters of the same patient were analyzed by Wilcoxon signed rank test. The Spearman rank correlation coefficient (r) was applied for the correlation analysis of laboratory parameters, and an r value less than −0.2 or greater than 0.2 was considered as a significant correlation. Risk factors for CT severity and prognosis were relatively determined through univariate and multivariate logistic regression analyses in which some laboratory parameters with significant correlations were not included. To analyze the diagnostic performance of independent risk factors, receiver operating characteristic (ROC) curves were constructed, the area under the curve (AUC)/concordance index (C-index) was calculated, and the LDH, neutrophil count, human serum amyloid A (SAA), and age with maximal values of Youden index were determined, respectively, as the optimal cutoff values. A 2-sided *p* < 0.05 was considered statistically significant. All analyses were performed with SPSS, version 19.0 (IBM SPSS), or R software, version 4.2.0 (R Foundation for Statistical Computing).

## Results

### Demographics and clinical characteristics

Data were collected in consecutive patients hospitalized with COVID-19. A total of 101 patients were included in this study (**Table 1**) after two patients were excluded because of acute myocardial infarction on admission and 15 patients were excluded due to mixed infections (nine cases with *Mycoplasma* pneumoniae, one with IBV, two with IBV + *Mycoplasma* pneumoniae mixed infections, three with ECHO virus). The median age was 72.00 years (IQR, 61.50–80.00 years), and 61 (60.4%) were men. According to the features of chest CT on admission, 51 (50.5%) of them were severe cases, and 50 (49.5%) were ordinary pneumonia cases. A total of 74 patients underwent two chest CT scans during hospitalization with a median interval time of 8.00 days (IQR, 6.00–9.00 days). Relief of pneumonia was shown on the second chest CT in 50 (67.6%) patients and was less common in severe cases (*p* = 0.007).

**Table 1:** Characteristics between ordinary and severe pneumonia groups.

Parameter	Total	Ordinary group	Severe group	*p* value
	(n=101)	(n=50)	(n=51)	
Age, median (IQR), y	72.00 (61.50-80.00)	68.00 (51.25-78.75)	75.00 (66.00-80.00)	0.044
Male, No. (%)	61 (60.4)	26 (52.0)	35 (68.6)	0.088
BMI, mean (SD), kg/m^2^	24.10 (4.08)	23.97 (3.95)	24.26 (4.27)	0.751
Onset of symptoms to first CT, median (IQR), d	7.00 (4.00-10.00)	5.50 (3.00-10.00)	7.00 (5.00-10.00)	0.048
Symptoms, No. (%)				
Fever	92 (91.1)	45 (90.0)	47 (92.2)	0.741
Cough	83 (82.2)	39 (78.0)	44 (86.3)	0.277
Expectoration	69 (68.3)	32 (64.0)	37 (72.5)	0.356
Pharyngalgia	16 (15.8)	13 (26.0)	3 (5.9)	0.007
Chest discomfort	35 (34.7)	19 (38.0)	16 (31.4)	0.484
Dyspnea	40 (39.6)	17 (34.0)	23 (45.1)	0.254
Palpitation	3 (3.0)	3 (6.0)	0 (0.0)	0.118
Nausea	12 (11.9)	8 (16.0)	4 (7.8)	0.234
Vomiting	7 (6.9)	5 (10.0)	2 (3.9)	0.269
Diarrhea	5 (5.0)	5 (10.0)	0 (0.0)	0.027
Poor appetite	61 (60.4)	27 (54.0)	34 (66.7)	0.193
Headache	10 (9.9)	6 (12.0)	4 (7.8)	0.525
Muscle soreness	17 (16.8)	13 (26.0)	4 (7.8)	0.018
Comorbidities, No. (%)				
Hypertension	45 (44.6)	22 (44.0)	23 (45.1)	0.912
Coronary heart disease	32 (31.7)	15 (30.0)	17 (33.3)	0.719
Diabetes	23 (22.8)	12 (24.0)	11 (21.6)	0.771
Chronic obstructive pulmonary disease	8 (7.9)	2 (4.0)	6 (11.8)	0.269
Old cerebral infarction	12 (11.9)	3 (6.0)	9 (17.6)	0.122
Malignant neoplasm	7 (6.9)	2 (4.0)	5 (9.8)	0.436
Blood routine				
White blood cell count, median (IQR), ×10^9^/L	5.88 (4.28-8.14)	5.00 (3.38-6.67)	7.04 (4.99-9.64)	0.001
Hemoglobin, mean (SD), g/L	127.49 (22.36)	128.38 (22.32)	126.61 (22.59)	0.693
Platelet count, median (IQR), ×10^9^/L	177.00 (139.50-235.50)	174.00 (139.75-213.75)	184.00 (139.00-282.00)	0.372
Lymphocyte count, median (IQR), ×10^9^/L	0.82 (0.57-1.56)	0.91 (0.58-1.22)	0.70 (0.53-1.12)	0.169
Neutrophil count, mean (SD), ×10^9^/L	5.04 (3.14)	3.85 (1.98)	6.20 (3.61)	<0.001
Inflammatory biomarkers				
Lactic dehydrogenase, median (IQR), U/L	259.30 (214.62-322.52)	234.10 (187.43-268.24)	291.92 (260.39-375.06)	<0.001
C-reactive protein, median (IQR), mg/L	45.84 (14.83-96.78)	18.79 (4.02-50.89)	72.35 (31.18-122.47)	<0.001
Human serum amyloid A, median (IQR), mg/L	243.74 (37.91-500.93)	83.26 (15.38-391.79)	448.69 (85.68-597.17)	0.003
Procalcitonin, median (IQR), ng/mL	0.09 (0.07-0.16)	0.08 (0.05-0.14)	0.11 (0.07-0.32)	0.102
Interleukin-6, median (IQR), pg/mL	36.75 (4.68-110.60)	14.25 (3.95-58.35)	110.60 (15.93-298.35)	0.095
Cardiac biomarkers				
HS-TnT, median (IQR), pg/mL	12.62 (6.97-21.00)	9.66 (5.09-15.75)	13.90 (8.80-26.90)	0.036
CKMB_M, median (IQR), ng/mL	1.24 (0.67-2.59)	1.04 (0.61-2.40)	1.46 (0.77-2.73)	0.248
Myoglobin, median (IQR), ng/mL	33.40 (20.00-85.40)	31.07 (20.00-74.21)	42.35 (27.85-102.29)	0.052
NT-proBNP, median (IQR), pg/mL	288.60 (146.40-770.00)	168.00 (34.83-528.75)	394.00 (174.50-1153.00)	0.016
Serum electrolytes				
Sodium, mean (SD), mmol/L	135.44 (5.58)	135.27 (6.63)	135.61 (4.39)	0.765
Potassium, mean (SD), mmol/L	3.97 (0.53)	4.05 (0.48)	3.90 (0.57)	0.165
Albumin, mean (SD), g/L	34.83 (5.72)	37.53 (4.97)	32.18 (5.18)	<0.001
Coagulation profiles				
D-dimer, median (IQR), mg/L	0.70 (0.40-1.23)	0.50 (0.31-0.88)	0.90 (0.52-1.69)	0.002
Fibrinogen, median (IQR), g/L	4.22 (3.53-4.95)	3.97 (3.34-4.62)	4.57 (3.96-5.74)	0.002

IQR, interquartile range; SD, standard deviation; BMI, body mass index; CT, computed tomography; HS-TnT, high-sensitivity troponin T; CKMB_M, creatine kinase–MB mass; NT-proBNP, N-terminal pro-brain natriuretic peptide.

The most commonly self-reported symptoms on admission were fever [n = 92 (91.1%)], cough [n = 83 (82.2%)], expectoration [n = 69 (68.3%)], poor appetite [n = 61 (60.4%)], dyspnea [n = 40 (39.6%)], and chest discomfort [n = 35 (34.7%)]. Ordinary pneumonia patients had significantly higher rates of symptoms including pharyngalgia [13 (26.0%) vs. 3 (5.9%); *p* = 0.007], diarrhea [5 (10.0%) vs. 0 (0.0%); *p* = 0.027], and muscle soreness [13 (26.0%) vs. 4 (7.8%); *p* = 0.018] (**Table 1**).

Among the 101 patients, 68 (67.3%) patients had comorbidities, including hypertension [n = 45 (44.6%)], coronary heart disease [n = 32 (31.7%)], diabetes [n = 23 (22.8%)], old cerebral infarction [n = 12 (11.9%)], chronic obstructive pulmonary disease [n = 8 (7.9%)], and malignant neoplasm [n = 7 (6.9%)] (**Table 1**). Rates of these comorbidities did not differ between severe pneumonia cases and ordinary pneumonia cases (**Table 1**) and between remission patients and non-remission patients (**Table 2**).

**Table 2:** Characteristics between pneumonia remission and non-remission groups.

Parameter	Remission group	Non-remission group	*p* value
	(n=50)	(n=24)	
Age, median (IQR), y	73.00 (62.25-81.00)	77.00 (65.25-80.00)	0.290
Male, No. (%)	31 (62.0)	14 (58.3)	0.762
BMI, mean (SD), kg/m^2^	25.09 (3.77)	23.82 (4.33)	0.257
Severe group, No. (%)	23 (46.0)	19 (79.2)	0.007
Interval time between two chest CT, median (IQR), d	7.50 (5.75-9.00)	8.00 (7.00-10.00)	0.119
Comorbidities, No. (%)			
Hypertension	24 (48.0)	11 (45.8)	0.861
Coronary heart disease	15 (30.0)	10 (41.7)	0.321
Diabetes	12 (24.0)	4 (16.7)	0.558
Chronic obstructive pulmonary disease	4 (8.0)	2 (8.3)	1.000
Old cerebral infarction	5 (10.0)	5 (20.8)	0.202
Malignant neoplasm	3 (6.0)	3 (12.5)	0.382
Blood routine			
White blood cell count, mean (SD), ×10^9^/L	5.46 (2.34)	7.82 (4.30)	0.017
Hemoglobin, mean (SD), g/L	130.80 (18.41)	124.38 (26.99)	0.233
Platelet count, median (IQR), ×10^9^/L	188.00 (148.75-232.25)	161.50 (110.00-291.00)	0.419
Lymphocyte count, median (IQR), ×10^9^/L	0.90 (0.59-1.36)	0.61 (0.39-0.84)	0.002
Neutrophil count, median (IQR), ×10^9^/L	3.57 (2.16-5.58)	5.90 (3.83-8.87)	0.003
Inflammatory biomarkers			
Lactic dehydrogenase, median (IQR), U/L	252.62 (209.90-289.19)	320.84 (250.55-381.67)	0.046
C-reactive protein, mean (SD), mg/L	51.19 (39.05)	89.96 (69.08)	0.021
Human serum amyloid A, median (IQR), mg/L	131.83 (37.91-461.26)	491.24 (229.63-651.04)	0.016
Procalcitonin, median (IQR), ng/mL	0.08 (0.05-0.11)	0.13 (0.09-0.36)	0.002
Interleukin-6, median (IQR), pg/mL	20.65 (3.52-46.10)	103.23 (63.73-471.75)	0.181
Cardiac biomarkers			
HS-TnT, median (IQR), pg/mL	10.95 (6.78-13.96)	17.66 (11.04-56.84)	0.009
CKMB_M, median (IQR), ng/mL	1.06 (0.65-1.64)	2.57 (1.27-4.57)	0.001
Myoglobin, median (IQR), ng/mL	30.90 (20.00-66.20)	70.04 (32.82-138.68)	0.005
NT-proBNP, median (IQR), pg/mL	169.00 (53.60-659.00)	597.55 (239.08-1871.75)	0.005
Serum electrolytes			
Sodium, mean (SD), mmol/L	136.59 (4.23)	134.00 (6.06)	0.068
Potassium, mean (SD), mmol/L	3.89 (0.48)	3.93 (0.60)	0.748
Albumin, mean (SD), g/L	35.76 (4.71)	32.65 (4.77)	0.010
Coagulation profiles			
D-dimer, median (IQR), mg/L	0.60 (0.39-1.11)	0.96 (0.60-2.09)	0.021
Fibrinogen, median (IQR), g/L	4.35 (3.97-5.00)	4.22 (3.65-5.75)	0.894
Therapy, No. (%)			
Glucocorticoid	49 (98.0)	21 (87.5)	0.097
Antibiotic	50 (100.0)	22 (91.7)	0.102
Antivirus			
Paxlovid	24 (48.0)	11 (45.8)	0.871
Azvudine	22 (44.0)	10 (41.7)	
Sivelestat sodium hydrate	4 (8.0)	5 (20.8)	0.139

IQR, interquartile range; SD, standard deviation; BMI, body mass index; CT, computed tomography; HS-TnT, high-sensitivity troponin T; CKMB_M, creatine kinase–MB mass; NT-proBNP, N-terminal pro-brain natriuretic peptide.

Glucocorticoid, antibiotic, and antivirus were the main treatment approaches for the hospitalized patients (**Table 2**). Of 74 patients, 35 (47.3%) received Paxlovid and 32 (43.2%) received azvudine with no significant difference between remission patients and non-remission patients (*p* = 0.871). Sivelestat sodium hydrate was given to nine (12.2%) patients, of whom five (55.6%) patients had no improvement in pneumonia after therapy.

### Laboratory parameters

Compared with ordinary pneumonia patients, patients in the severe group presented with significantly higher white blood cell (WBC) count, neutrophil count, LDH, C-reactive protein (CRP), SAA, high-sensitivity troponin T (HS-TnT), N-terminal pro-brain natriuretic peptide (NT-proBNP), D-dimer, and fibrinogen and lower albumin (*p* < 0.05 for all) (**Table 1**).

Compared with remission patients, non-remission patients presented with significantly higher WBC count, neutrophil count, LDH, CRP, SAA, procalcitonin (PCT), HS-TnT, creatine kinase–MB mass (CKMB_M), myoglobin, NT-proBNP, and D-dimer and lower lymphocyte count and albumin on admission (*p* < 0.05 for all) (**Table 2**).

**Figure 2:**
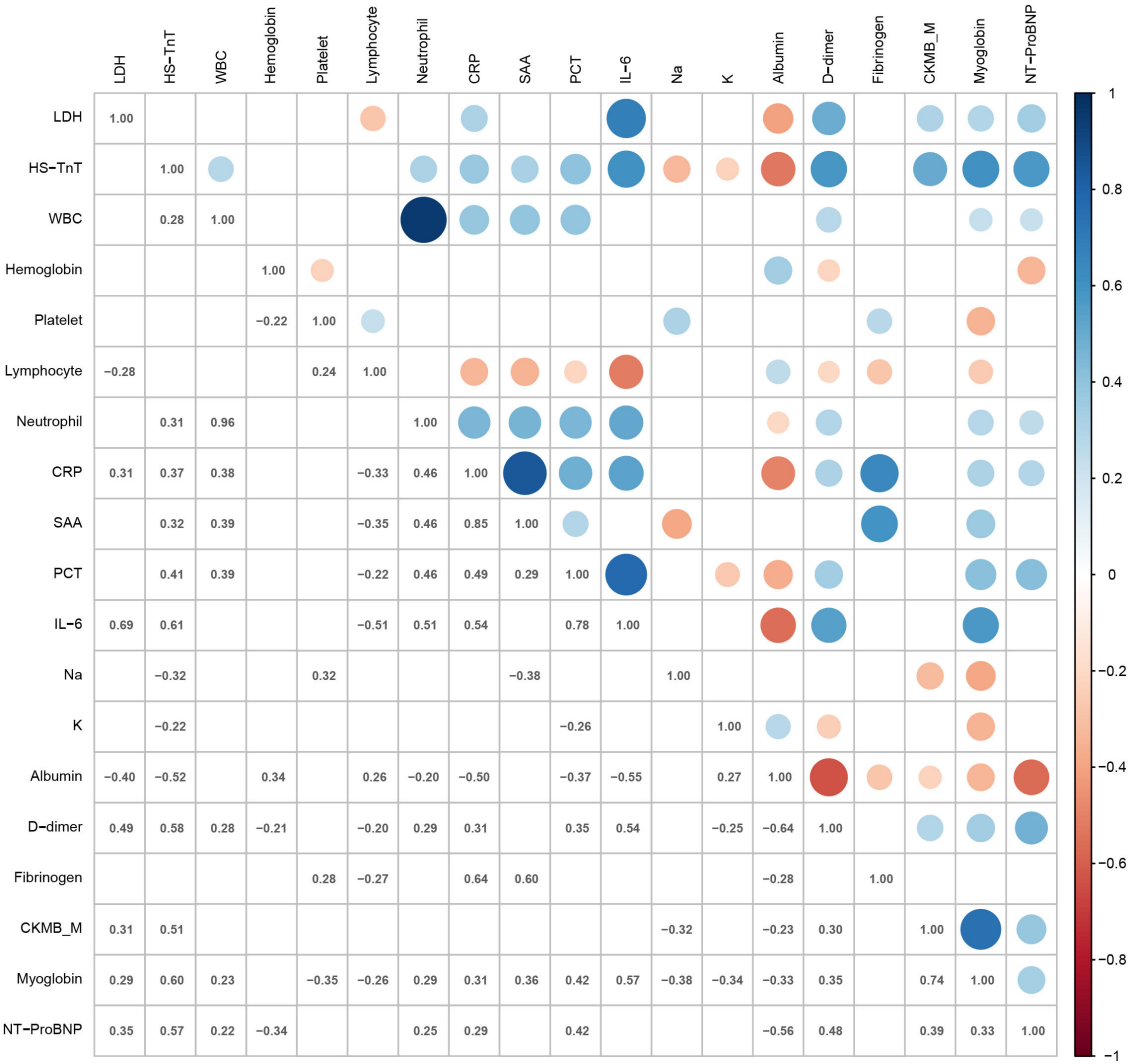
Heat map representing the correlation of laboratory parameters. LDH, lactic dehydrogenase; HS-TnT, high-sensitivity troponin T; WBC, white blood cell count; Platelet, platelet count; Lymphocyte, lymphocyte count; Neutrophil, neutrophil count; CRP, C-reactive protein; SAA, human serum amyloid A; PCT, procalcitonin; IL-6, interleukin-6; Na, sodium; K, potassium; CKMB_M, creatine kinase–MB mass; NT-proBNP, N-terminal pro-brain natriuretic peptide.

The correlation of laboratory parameters is shown in **Figure 2**. LDH levels in patients with COVID-19 correlated significantly with CRP (ρ = 0.31, *p* = 0.024), albumin (ρ = -0.40, *p* = 0.002), CKMB_M (ρ = 0.31, *p* = 0.028), and myoglobin (ρ = 0.29, *p* = 0.036). The neutrophil count showed strong positive correlations with the WBC count (ρ = 0.96, *p* < 0.001). HS-TnT levels correlated significantly with CRP (ρ = 0.37, *p* = 0.001), SAA (ρ = 0.32, *p* = 0.022), sodium (ρ = -0.32, *p* = 0.004), albumin (ρ = -0.52, *p* < 0.001), CKMB_M (ρ = 0.51, *p* < 0.001), and myoglobin (ρ = 0.60, *p* < 0.001). SAA showed positive correlations with CRP (ρ = 0.85, *p* < 0.001) and fibrinogen (ρ = 0.60, *p* < 0.001).

### Risk factors for CT severity and prognosis

**Figure 3:**
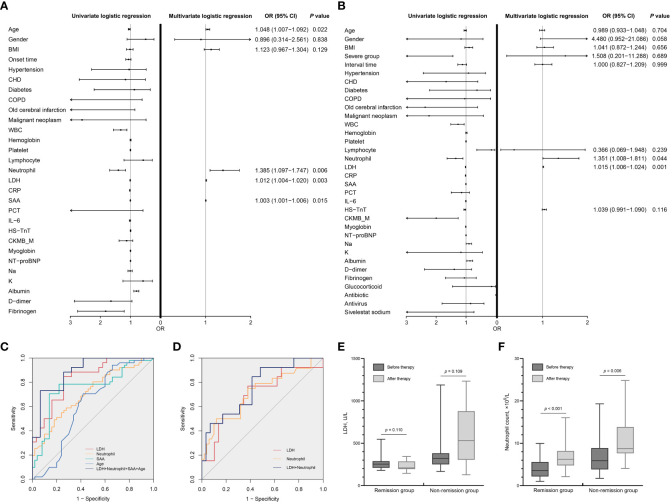
Analysis of risk factors for CT severity and prognosis. **(A)** Univariate and multivariate logistic regression analyses of risk factors associated with CT severity. **(B)** Univariate and multivariate logistic regression analyses of risk factors associated with prognosis. The OR and 95% CI of each factor are presented as points and lines, respectively, in **(A, B)**. **(C)** ROC curves for predicting CT severity on admission (**Table 3**). **(D)** ROC curves for predicting the prognosis of COVID-19 patients (**Table 3**). **(E)** Dynamic changes of LDH during hospitalization. **(F)** Dynamic changes of neutrophil count during hospitalization. CT, computed tomography; OR, odds ratio; CI, confidence interval; ROC, receiver operating characteristic; COVID-19, coronavirus disease 2019; BMI, body mass index; Onset time, onset of symptoms to first CT; CHD, coronary heart disease; COPD, chronic obstructive pulmonary disease; WBC, white blood cell count; Platelet, platelet count; Lymphocyte, lymphocyte count; Neutrophil, neutrophil count; LDH, lactic dehydrogenase; CRP, C-reactive protein; SAA, human serum amyloid A; PCT, procalcitonin; IL-6, interleukin-6; HS-TnT, high-sensitivity troponin T; CKMB_M, creatine kinase–MB mass; NT-proBNP, N-terminal pro-brain natriuretic peptide; Na, sodium; K, potassium; Interval time, interval time between two chest CTs.

Univariate logistic regression analysis showed that age, WBC count, neutrophil count, LDH, CRP, SAA, albumin, and fibrinogen were significantly associated with CT severity at admission (all *p* < 0.05). Multivariate logistic regression analysis showed that age [odds ratio (OR): 1.048; 95% confidence interval (CI): 1.007−1.092; *p* = 0.022], LDH (OR: 1.012; 95% CI: 1.004−1.020; *p* = 0.003), neutrophil count (OR: 1.385; 95% CI: 1.097−1.747; *p* = 0.006), and SAA (OR: 1.003; 95% CI: 1.001−1.006; *p* = 0.015) were independent risk factors for CT severity at admission (**Figure 3A**; **Table 3**).

**Table 3:** Predictive ability of independent risk factors on CT severity and prognosis.

Parameter	Cutoff value	AUC (95%CI)	Sensitivity (%)	Specificity (%)	*p* value
Severity of pneumonia shown on the first chest CT					
LDH	256.69 U/L	0.833 (0.729-0.936)	84.6	74.2	<0.001
Neutrophil	6.03 ×10^9^/L	0.711 (0.611-0.810)	49.0	82.0	<0.001
SAA	462.23 mg/L	0.718 (0.592-0.844)	48.5	86.7	0.003
Age	69.50 y	0.616 (0.503-0.730)	70.6	58.0	0.044
LDH+Neutrophil+SAA+Age		0.891 (0.809-0.972)	73.1	93.5	<0.001
Remission of pneumonia shown on the second chest CT					
LDH	258.46 U/L	0.695 (0.514-0.876)	76.9	62.1	0.046
Neutrophil	6.76 ×10^9^/L	0.713 (0.582-0.844)	50.0	88.0	0.003
LDH+ Neutrophil		0.759 (0.603-0.914)	92.3	51.7	0.008

AUC, area under the curve; CT, computed tomography; LDH, lactic dehydrogenase; Neutrophil, neutrophil count; SAA, human serum amyloid A.

Univariate logistic regression analysis showed that severe group, WBC count, lymphocyte count, neutrophil count, LDH, CRP, SAA, HS-TnT, albumin, CKMB_M, myoglobin, and sodium were significantly associated with remission of pneumonia (all *p* < 0.05). Multivariate logistic regression analysis showed that LDH (OR: 1.015; 95% CI: 1.006–1.024; *p* = 0.001) and neutrophil count (OR: 1.351; 95% CI: 1.008–1.811; *p* = 0.044) were independent risk factors for remission of pneumonia (**Figure 3B**; **Table 3**).

### Predictive ability of independent risk factors on CT severity and prognosis

For ROC analysis, the AUC of LDH was 0.833 (95% CI: 0.729–0.936; *p* < 0.001), of neutrophil count was 0.711 (95% CI: 0.611–0.810; *p* < 0.001), of SAA was 0.718 (95% CI: 0.592–0.844; *p* = 0.003), and of age was 0.616 (95% CI: 0.503–0.730; *p* = 0.044) for the ordinary pneumonia and severe pneumonia patients’ discrimination. The optimal cutoff value for LDH levels was 256.69 U/L (specificity 84.6%, sensitivity 74.2%), for neutrophil count was 6.03 × 10^9^/L (specificity 49.0%, sensitivity 82.0%), for SAA was 462.23 mg/L (specificity 48.5%, sensitivity 86.7%), and for age was 69.50 years (specificity 70.6%, sensitivity 58.0%) (**Figure 3C**; **Table 3**). The optimal cutoff point for LDH and neutrophil count was 258.46 U/L (AUC = 0.695, specificity 76.9%, sensitivity 62.1%) and 6.76 × 10^9^/L (AUC = 0.713, specificity 50.0%, sensitivity 88.0%), respectively, at discriminating remission and non-remission patients (**Figure 3D**; **Table 3**).

### Dynamic changes of LDH and neutrophil count levels during hospitalization

**Figures 3E, F** show the dynamic changes of LDH and neutrophil count levels for remission and non-remission patients during hospitalization. LDH levels decreased in remission patients [median (IQR), 252.62 (209.90–289.19) U/L vs. 209.14 (195.22–285.01) U/L; *p* = 0.110] and increased in non-remission patients [median (IQR), 320.84 (250.55–381.67) U/L vs. 531.20 (304.47–878.35) U/L; *p* = 0.109], but the difference was not evident. Neutrophil count levels increased significantly in remission patients [median (IQR), 3.57 (2.16–5.58) pg/mL vs. 6.21 (4.78–8.19) pg/mL; *p* < 0.001] and also increased in non-remission patients [median (IQR), 5.90 (3.83–8.87) pg/mL vs. 8.74 (7.63–13.86) pg/mL; *p* = 0.006].

## Discussion

The pneumonia caused by the novel coronavirus infection COVID-19 is a public health event that seriously endangers people’s lives and health. According to public data of previous studies, approximately 8%–30% of patients would eventually develop severe illness and approximately 1%–11% of patients would die (Huang et al., 2020). As a new infectious disease, the lack of overall cognition makes it difficult for clinicians to conduct timely and accurate diagnosis and treatment. Our analysis was focused on identifying high-risk COVID-19 patients developing severe illness and will help clinicians in identifying potentially critical patients earlier, allocating medical resources more reasonably, paying more attention to these patients, thus giving necessary interventions as early as possible. In contrast to earlier findings in COVID-19, our study reinforced the longitudinal variation in the biomarkers, which may be a more comprehensive assessment for the patient’s conditions.

With a serious SARS-CoV-2 infection, the host immune system becomes overactivated and even out of control. Many cytokines are secreted to eliminate the virus. When SARS-CoV-2 infection caused an “inflammatory storm,” not only associated cytokines rise sharply but some inflammatory biomarkers also increase, such as CRP, serum ferritin, SAA, and PCT. This retrospective analysis is consistent with previous reports that D-dimer, leukocyte count, neutrophil count, CRP, and SAA indicate a high risk of severe illness and poor prognosis of COVID-19 (Chen et al., 2020; Li H. et al., 2020; Qin et al., 2020; Wang et al., 2020; Zhou et al., 2020).

Glycolysis is a metabolic pathway that produces energy for biological functions. Glycolysis involves a set of enzymes that convert glucose to pyruvate. LDH is one of the enzymes that catalyze the interconversion of pyruvate and lactate, which is a crucial step in the anaerobic metabolism of glucose when oxygen is unavailable or limited (Theret et al., 2017). As an important glycolytic pathway enzyme, LDH is widely distributed in myocardium, liver, kidney, and lung tissues (Livesey et al., 2020). And its concentration in tissues is much higher than that in serum. When these tissues have ischemia and hypoxia necrosis, a large amount of LDH can be released, resulting in a sharp increase in serum levels. COVID-19 patients have respiratory dysfunction and extensive inflammatory damage in tissues and organs. When immune cells are triggered to create an inflammatory response, they also undergo metabolic reprogramming. The tissues and organs are in the state of hypercapnia and hypoxia for a long time, which can cause mitochondrial dysfunction and adenosine triphosphate (ATP) synthesis decline, and then lead to sodium pump dysfunction, cell swelling, and necrosis, resulting in increased LDH release. The damage of myocardial cells caused by the inflammatory reaction or serious infection can also lead to a large amount of LDH release and then cause the increase of serum LDH. The high level of LDH means the serious damage of heart, lung, and other tissues. Among the risk variables, LDH exhibits the strongest direct relationship with both the P/F ratio and the CT score in the lungs, indicating a clear link between lung damage and disease severity (Ding et al., 2017; Wu et al., 2017; Mo et al., 2018). It is reported to be one closely related biomarker with the mortality rate of ARDS (Hoeboer et al., 2015).

An elevated LDH is a common manifestation in patients infected with respiratory syndrome coronavirus in the Middle East (MERS CoV) (Assiri et al., 2013; Al Ghamdi et al., 2016) and H7N9 (Shi et al., 2013; Liu et al., 2021). In this study, LDH levels in the severe pneumonia group were significantly higher than those in the ordinary pneumonia group, and the LDH level in the pneumonia remission group was lower than that in the non-remission group. The ROC curve suggests that the sensitivity is 76.9% and the specificity is 62.1% to predict severe pneumonia when the LDH level is higher than 258.46 U/L. In recent years, LDH elevations have been described in fatal cases from multiorgan system failure (Perez-Padilla et al., 2009), and the continuous and significant increase of LDH can indicate the poor prognosis of COVID-19 (Rodrigues et al., 2021). Laguna-Goya et al (Rocio et al., 2020). found that the blood LDH level of COVID-19 patients increased with the prolongation of virus infection time and the aggravation of disease. In this study, the LDH level was increased in patients with poor prognosis of COVID-19, suggesting that the LDH value in serum was associated with the severity of clinical pneumonia and can predict the severity of disease in patients with COVID-19 pneumonia. Moreover, neutrophils act as the first cellular defense against infection (Kolaczkowska and Kubes, 2013). Neutrophil count is an important indicator for evaluating the severity of inflammation. In the current study, it was interesting that the combined detection of LDH and neutrophil count was more sensitive in predicting severe pneumonia than the single neutrophil count.

In conclusion, the level of LDH initial values in COVID-19 patients can be used as a warning indicator for the severity of the disease. Therefore, the detection of serum LDH can be used as an important index to evaluate the severity of disease progression and prognosis of COVID-19 patients. This study is a single-center retrospective study, and we need to increase the sample number to support our conclusion and include some special examination results, disease development, and laboratory dynamic results and the elements related to disease severity, complications, and fatality rate for a comprehensive analysis.

## Data availability statement

The original contributions presented in the study are included in the article/supplementary material. Further inquiries can be directed to the corresponding author.

## Ethics statement

The studies involving humans were approved by biomedical research ethic committee of shandong provincial hospital. The studies were conducted in accordance with the local legislation and institutional requirements. The participants provided their written informed consent to participate in this study.

## Author contributions

JT conceived the project, designed the experiments, and wrote the paper; TM and XW collected patient samples and analyzed data;ZL provided the statistical support. All authors contributed to the article and approved the submitted version.

## References

[B1] Al Ghamdi M. Alghamdi K. M. Ghandoora Y. Alzahrani A. Salah F. Alsulami A.. (2016). Treatment outcomes for patients with middle eastern respiratory syndrome coronavirus (mers cov) infection at a coronavirus referral center in the Kingdom of Saudi Arabia. BMC Infect. Dis. 16. doi: 10.1186/s12879-016-1492-4 PMC4839124 27097824

[B2] Assiri A. Al-Tawfiq J. A. Al-Rabeeah A. A. Al-Rabiah F. A. Al-Hajjar S. Al-Barrak A.. (2013). Epidemiological, demographic, and clinical characteristics of 47 cases of middle east respiratory syndrome coronavirus disease from Saudi Arabia: a descriptive study. Lancet Infect. Dis 13, 752–761. doi: 10.1016/S1473-3099(13)70204-4 23891402 PMC7185445

[B3] Chen G. Wu D. Guo W. Cao Y. Huang D. Wang H.. (2020). Clinical and immunological features of severe and moderate coronavirus disease 2019. J. Clin. Invest 130, 2620–2629. doi: 10.1172/JCI137244 32217835 PMC7190990

[B4] Ding J. Karp J. E. Emadi A. (2017). Elevated lactate dehydrogenase (ldh) can be a marker of immune suppression in cancer: interplay between hematologic and solid neoplastic clones and their microenvironments. Cancer biomark. 19, 353–363. doi: 10.3233/CBM-160336 28582845 PMC13020749

[B5] Hoeboer S. H. Straaten H. M. O. Groeneveld A. J. (2015). Albumin rather than c-reactive protein may be valuable in predicting and monitoring the severity and course of acute respiratory distress syndrome in critically ill patients with or at risk for the syndrome after new onset fever. BMC Pulm Med. 15. doi: 10.1186/s12890-015-0015-1 PMC4381515 25888398

[B6] Huang C. Wang Y. Li X. Ren L. Zhao J. Hu Y.. (2020). Clinical features of patients infected with 2019 novel coronavirus in wuhan, China. Lancet 395, 497–506. doi: 10.1016/S0140-6736(20)30183-5 31986264 PMC7159299

[B7] Kevadiya B. D. Machhi J. Herskovitz J. Oleynikov M. D. Blomberg W. R. Bajwa N.. (2021). Diagnostics for SARS-cov-2 infections. Nat. Mater. 20, 593–605. doi: 10.1038/s41563-020-00906-z 33589798 PMC8264308

[B8] Kolaczkowska E. Kubes P. (2013). Neutrophil recruitment and function in health and inflammation. Nat. Rev. Immunol. 13, 159–175. doi: 10.1038/nri3399 23435331

[B9] Li H. Xiang X. Ren H. Xu L. Zhao L. Chen X.. (2020). Serum Amyloid A is a biomarker of severe Coronavirus Disease and poor prognosis. J. Infect. 80, 646–655. doi: 10.1016/j.jinf.2020.03.035 32277967 PMC7141628

[B10] Li K. Wu J. Wu F. Guo D. Chen L. Fang Z.. (2020). The clinical and chest ct features associated with severe and critical covid-19 pneumonia. Invest. Radiol. 55, 327–331. doi: 10.1097/RLI.0000000000000672 32118615 PMC7147273

[B11] Libby P. (2020). The heart in covid19 primary target or secondary bystander. JACC Basic Transl. Sci. 20, 537–542. doi: 10.1016/j.jacbts.2020.04.001 PMC7151324 32292847

[B12] Liu W. J. Xiao H. Dai L. Liu D. Chen J. Qi X.. (2021). Avian influenza a (h7n9) virus: from low pathogenic to highly pathogenic. Front. Med-Prc 15, 507–527. doi: 10.1007/s11684-020-0814-5 PMC8190734 33860875

[B13] Livesey A. Garty F. Shipman A. R. Shipman K. E. (2020). Lactate dehydrogenase in dermatology practice. Clin. Exp. Dermatol. 45, 539–543. doi: 10.1111/ced.14134 31755143

[B14] Mo X. Wei F. Tong Y. Ding L. Zhu Q. Du S.. (2018). Lactic acid downregulates viral microrna to promote epstein-barr virus-immortalized b lymphoblastic cell adhesion and growth. J. Virol. 92, e00033–18. doi: 10.1128/JVI.00033-18 29444941 PMC5899195

[B15] Perez-Padilla R. de la Rosa-Zamboni D. Ponce D. L. S. Hernandez M. Quinones-Falconi F. Bautista E.. (2009). Pneumonia and respiratory failure from swine-origin influenza a (h1n1) in Mexico. N. Engl. J. Med. 361, 680–689. doi: 10.1056/NEJMoa0904252 19564631

[B16] Qin C. Zhou L. Hu Z. Zhang S. Yang S. Tao Y.. (2020). Dysregulation of immune response in patients with coronavirus 2019 (Covid-19) in Wuhan, China. Clin. Infect. Dis. 71, 762–768. doi: 10.1093/cid/ciaa248 32161940 PMC7108125

[B17] Rocio L. Alberto U. Paloma T. Maria L. Angel R. Laura N.. (2020). Il-6-based mortality risk model for hospitalized patients with covid-19. J. Allergy Clin. Immunol. 146 (4), 799–807. doi: 10.1016/j.jaci.2020.07.009 32710975 PMC7375283

[B18] Rodrigues T. S. de Sá K. S. G. Ishimoto A. Y. Becerra A. Oliveira S. Almeida L.. (2021). Inflammasomes are activated in response to SARS-cov-2 infection and are associated with covid-19 severity in patients. J. Exp. Med. 218, e20201707. doi: 10.1084/jem.20201707 33231615 PMC7684031

[B19] Shi J. Xie J. He Z. Hu Y. He Y. Huang Q.. (2013). A detailed epidemiological and clinical description of 6 human cases of avian-origin influenza a (h7n9) virus infection in Shanghai. PloS One 8, e77651. doi: 10.1371/journal.pone.0077651 24143251 PMC3797049

[B20] Theret M. Gsaier L. Schaffer B. Juban G. Ben L. S. Weiss-Gayet M.. (2017). Ampkalpha1-ldh pathway regulates muscle stem cell self-renewal by controlling metabolic homeostasis. EMBO J. 36, 1946–1962. doi: 10.15252/embj.201695273 28515121 PMC5494470

[B21] Wang D. Hu B. Hu C. Zhu F. Liu X. Zhang J.. (2020). Clinical characteristics of 138 hospitalized patients with 2019 novel coronavirus- infected pneumonia in Wuhan, China. JAMA 17, 1061–1069. doi: 10.1001/jama.2020.1585 PMC7042881 32031570

[B22] Wu Y. Cui X. Wu N. Song R. Yang W. Zhang W.. (2017). A unique case of human zika virus infection in association with severe liver injury and coagulation disorders. Sci. Rep-Uk 7, 11393. doi: 10.1038/s41598-017-11568-4 PMC5595821 28900143

[B23] Zheng Y. Wang L. Ben S. (2021). Meta-analysis of chest ct features of patients with covid-19 pneumonia. J. Med. Virol. 93, 241–249. doi: 10.1002/jmv.26218 32579236 PMC7361361

[B24] Zhong-shu Kuang Y. Y. W. W. Sun Z. S. (2020). Clinical characteristics and prognosis of community- acquired pneumonia in autoimmune disease- induced immunocompromised host: a retrospective observational study. World J. Emerg. Med. 3, 145–151. doi: 10.5847/wjem.j.1920-8642.2020.03.003 PMC7183923 32351646

[B25] Zhou F. Yu T. Du R. Fan G. Liu Y. Liu Z.. (2020). Clinical course and risk factors for mortality of adult inpatients with covid-19 in Wuhan, China: a retrospective cohort study. Lancet 395, 1054–1062. doi: 10.1016/S0140-6736(20)30566-3 32171076 PMC7270627

